# Leucocyte telomere length, brain volume and risk of dementia: a prospective cohort study

**DOI:** 10.1136/gpsych-2023-101120

**Published:** 2023-09-11

**Authors:** Zhi Cao, Yabing Hou, Chenjie Xu

**Affiliations:** 1 School of Public Health, Hangzhou Normal University, Hangzhou, China; 2 School of Public Health, Zhejiang University School of Medicine, Hangzhou, China; 3 Yanjing Medical College, Capital Medical University, Beijing, China

**Keywords:** association, environmental psychology, neurocognitive disorders

## Abstract

**Background:**

The evidence regarding the association between leucocyte telomere length (LTL) and brain health is sparse and inconclusive.

**Aims:**

To investigate the associations of LTL with brain structure and the risk of dementia based on a large-scale prospective study.

**Methods:**

LTL in the peripheral blood was measured by the quantitative polymerase chain reaction (qPCR) assay from 439 961 individuals in the UK Biobank recruited between 2006 and 2010 and followed up until 2020. Electronic health records were used to record the incidence of dementia, including Alzheimer’s disease (AD) and vascular dementia (VD). The brain structure, including total and regional brain volume, of 38 740 participants was then assessed by magnetic resonance imaging (MRI).

**Results:**

During a median follow-up of 11.6 years, a total of 5 820 (1.3%) dementia cases were documented. The restricted cubic spline model showed significant overall associations between LTL and the risk of dementia and AD (p for overall <0.05). The multivariable adjusted hazard ratios (HRs) for the lowest LTL tertile compared with the highest LTL tertile were 1.14 (95% confidence interval (CI): 1.06 to 1.21) for dementia, 1.28 (95% CI: 1.12 to 1.46) for AD and 1.18 (95% CI: 0.98 to 1.42) for VD. Furthermore, we found that shorter LTL was associated with smaller total brain volume (β=−0.012 8, p=0.003), white matter volume (β=−0.022 4, p<0.001), hippocampus volume (β=−0.017 2, p<0.001), thalamus volume (β=−0.023 9, p<0.001) and accumbens (β=−0.015 5, p=0.001).

**Conclusions:**

Shorter LTL is associated with total and regional brain structure and a higher risk of incident dementia and AD, implying the potential of telomere length as a predictive biomarker of brain health.

**WHAT IS ALREADY KNOWN ON THIS TOPIC**

- Shorter telomere length was associated with a higher risk of dementia and Alzheimer’s disease (AD), although several studies showed inconsistent results.
- The evidence regarding the association between telomere length and dementia was mostly examined by Mendelian randomisation using genetic variation.
- Few studies have confirmed this association in large epidemiological cohort studies because of unavailable telomere length measurement.

**WHAT THIS STUDY ADDS**

- We found that shorter leucocyte telomere length (LTL) was associated with a higher risk of dementia and AD.
- We also observed that shorter LTL was associated with smaller total brain volume, white matter volume and subcortical brain structures (eg, thalamus, hippocampus, accumbens, putamen, pallidum), and greater white matter hyperintensity volumes.

**HOW THIS STUDY MIGHT AFFECT RESEARCH, PRACTICE OR POLICY**

- Our findings underscore a relationship between LTL and dementia, providing potential public and clinical applications.
- People who inherit shorter LTL may be predisposed to dementia, making LTL an effective biomarker for dementia prevention.

## Introduction

Dementia is one of the greatest challenges for health and social care in the 21st century due to the ageing of the population worldwide.^1 2^ The number of people living with dementia is projected to triple in the upcoming 30 years.^3^ Although advances in the past decades have revealed several pathological mechanisms underlying dementia, chronological age remains the most important risk factor for dementia.^4^ Telomeres are protein-DNA complexes at the end of chromosomes that prevent the loss of coding DNA, however, they lessen over time as the ends of chromosomes become shortened with every cell division.^5^ Telomere length has emerged as a promising biomarker of cellular ageing and an indicator of susceptibility to age-related diseases.^6^ Because the measurement of leucocyte telomere length (LTL) by quantitative polymerase chain reaction (qPCR) in large-scale population-based studies is impractical, current evidence has yet not supported LTL as a predictive biomarker in disease prevention and screening.

Several prior observational studies with small sample sizes (<3 000 participants) reported the association between shortening LTL and age-related diseases, such as mild cognitive impairment, dementia and Alzheimer’s disease (AD).^7–10^ However, the associations between LTL and these brain-related disorders remain inconsistent. For instance, findings from prospective studies showed that both short LTL and long LTL were associated with mild cognitive impairment and AD.^8 9^ In contrast, a retrospective study did not find any associations of LTL with dementia or mild cognitive impairment.^10^ Discrepancies in these findings might be attributed to different study designs and populations, lack of consideration of possible confounders and small sample sizes. In addition, magnetic resonance imagings (MRIs) of the brain in cross-sectional and longitudinal cohort investigations have demonstrated that brain atrophy is a highly sensitive indicator of mild cognitive impairment and dementia.^11^ And only a handful of studies have examined the effect of LTL on regional brain volume, such as in the hippocampus and thalamus.^12^ However, most brain studies that rely on MRI scans do not include sufficient participants to provide reliable results.^13^ Furthermore, little is known about the association between LTL and subcortical brain structures due to data inaccessibility in large-scale populations.

The UK Biobank is a large-scale database drawn from a prospective cohort study that covers 500 000 individuals. The recently released LTL data were measured from the DNA samples of 474 074 participants. This offers us a novel opportunity to systemically evaluate the correlation of LTL with brain health in a larger population-based sample. Based on the UK Biobank dataset, we aimed to examine the prospective association between LTL and the risk of dementia and to identify the linear associations of LTL with total and regional brain structures.

## Methods

### Study design and population

**Figure 1:**
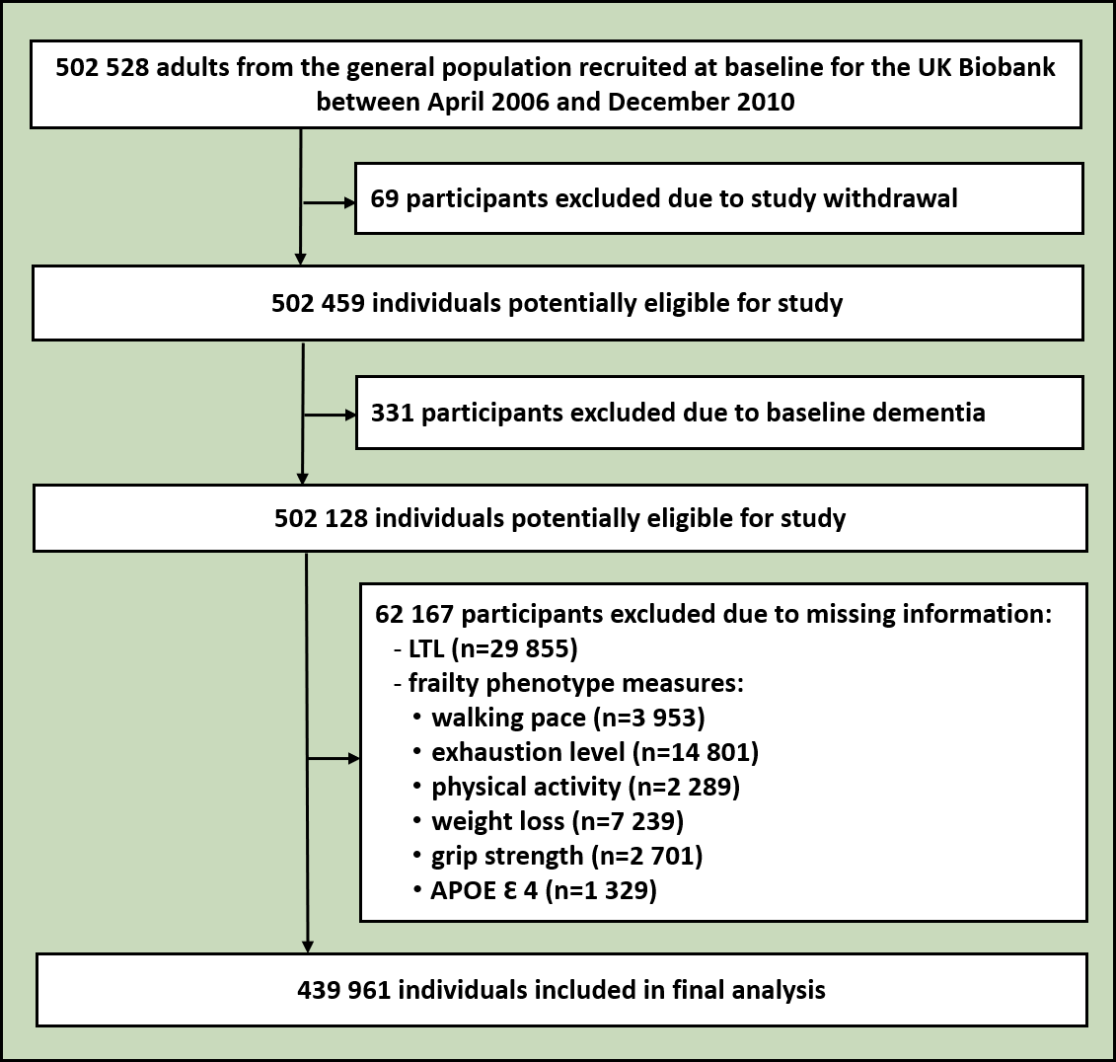
Flowchart of the study. APOE, apolipoprotein E; LTL, leucocyte telomere length.

This is a prospective, population-based cohort study of participants enrolled in the UK Biobank. Between April 2006 and December 2010, the UK Biobank recruited 502 528 adults (37–73 years old) from the general population in the UK. Participants attended one of the 22 assessment centres across England, Scotland and Wales to complete nurse-led electronic questionnaires, physical examinations and biological sample collections, and agree to long-term follow-up for multiple health-related outcomes.^14^ An imaging substudy was incorporated into the UK Biobank in 2014 that strove to include brain, heart and body MRI imaging from 100 000 participants.^15^ The LTL data for 439 961 participants free of dementia were used to assess the association between LTL and dementia, while brain volume analysis was conducted for 38 740 participants with valid data on brain imaging (figure 1). All participants gave written informed consent prior to data collection.

### LTL measurement

The genotype from peripheral blood leucocytes was extracted from the UK Biobank as part of a cohort-wide array.^16^ LTL was measured using qPCR assay, and results are reported as relative ratios of the copy number of the telomere DNA to a single-copy gene (T/S ratios).^17^ LTL used in this study was adjusted for the influence of technical parameters. The measurement of LTL in the UK Biobank participants and the extensive quality checks and adjustment for technical factors are reported by Codd *et al*.^18^

### Total and regional brain volume

A 3-Tesla, 32-channel coil Siemens Skyra scanner (Siemens Medical Solutions, Germany) was used by the UK Biobank to obtain the MRIs, with 1×1×1 resolution and a view field of 208×256×256. The MRI protocols have been described in detail elsewhere.^15^ Briefly, the numerical volume was calculated by preprocessed three-dimensional magnetisation for rapid echo-gradient (3D magnetization prepared-rapid gradient echo (MP-RAGE)) T1-weighted image-derived phenotypes. The T2-fluid-attenuated inversion recovery (FLAIR) structural imaging was undertaken in 6 min with TR 5 000.0 ms, TE 395.0 ms and a spatial resolution of 1.05×1×1 mm. Total brain, white matter, grey matter and subcortical brain structures were generated from the processed T1 images, while combined analyses of T1 and T2-FLAIR data quantified white matter hyperintensity volume. Total brain volume, white matter volume, grey matter volume and subcortical brain volume were noted in mm^3^ and normalised for head size. Full details on structural image segmentation and data normalisation are provided elsewhere.^19^

### Ascertainment of dementia

The primary disease outcomes for this study were all-cause dementia, AD and vascular dementia (VD). According to World Health Organization (WHO), dementia is defined as a syndrome—usually of a chronic or progressive nature—in which there is deterioration in cognitive function (ie, the ability to process thought) beyond what might be expected from normal ageing.^20^ Every resident in England, Scotland and Wales has a unique National Health Service (NHS) identification number, which was used for linking all participants to electronic health records. The diagnosis for incident all-cause dementia (F00–F03, G30–G31), AD (F00, G30) and VD (F01, I67.3) was coded according to the WHO International Classification of Diseases, Tenth Revision (ICD-10). The first known hospitalisation with relevant diagnostic codes post-recruitment was recorded. Participants were followed up from enrolment until the incidence of dementia, the date of death or the end of the follow-up (31 December 2020), whichever came first.

### Covariates

A wide range of sociodemographic, lifestyle and familial factors and chronic diseases were considered as potential confounders. Information on sex, ethnicity, educational attainment, smoking status, alcohol intake frequency and family history of dementia was collected from a touchscreen questionnaire. The Townsend deprivation score was used to measure socioeconomic status and was assigned to participants based on their residential postcode at recruitment.^21^ Body mass index (BMI) was calculated as weight (in kilograms) divided by height (in metres) squared. Hypertension was defined as systolic blood pressure (BP) ≥140 or diastolic BP ≥90 mm Hg or regular use of antihypertensive medication. Hypercholesterolaemia was defined as total cholesterol ≥6.2 mmol/L or regular use of lipid-lowering medication. Hyperglycaemia was defined as fasting blood glucose ≥7.0 mmol/L or regular use of antidiabetic medications. UK Biobank genotyping was conducted by Affymetrix using a bespoke BiLEVE Axiom array and the Affymetrix UK Biobank Axiom array. The apolipoprotein E (APOE) genotype was directly genotyped. Further information on covariates can be found (https://biobank.ctsu.ox.ac.uk/crystal/docs/genotyping_sample_workflow.pdf). The APOE ε4 carrier status was defined as carriers versus non-carriers of the ε4 allele. Depressive symptoms were assessed with the validated 2-item Patients Health Questionnaire (PHQ-2),^22^ and widely adjusted dementia-related comorbidities (self-reported type 2 diabetes and cardiovascular disease (CVD)) were additionally considered as potential confounders.

### Statistical analysis

The baseline characteristics of the LTL tertile were summarised using descriptive statistics. The mean and standard deviation (SD) of the continuous variables, and the number and proportion of each categorical variable were calculated. The χ^2^ test was used to compare categorical variables of the LTL tertile baseline characteristics, while the one-way analysis of variance test was applied to compare continuous variables.

Cox proportional hazard models with age as a timescale were used to estimate hazard ratios (HRs) and 95% confidence intervals (CIs) for the associations of LTL with the risk of incident dementia, AD and VD. The proportional hazard assumption was checked by tests based on Schoenfeld residuals, and the results indicated that the assumptions had not been violated. Restricted cubic spline models with five knots were used to investigate the shape of associations between LTL and dementia. Effect modification analyses were conducted to assess whether the association between LTL and dementia differed by familial factors, including APOE genotype and family history of dementia. Missing information on covariates was coded as a missing category for categorical variables, and with mean values for continuous variables, the missing percentage was very low (less than 1%).

General linear regression models were used to estimate the associations between LTL and brain volume. Multivariable analyses were adjusted for age, sex, ethnicity, educational attainment, the Townsend deprivation index score, smoking status, alcohol intake frequency, BMI, APOE ε4 status, family history of dementia, hypertension, hypercholesterolaemia and hyperglycaemia. The differences in brain volume between age and sex subgroup were also examined. Interactions between LTL and the APOE genotype were also tested by adding LTL×APOE ε4 terms to the models.

Several sensitivity analyses were carried out to reinforce the robustness of the results. First, considering the strong relationship between age and dementia, we fitted age-constant survival models to determine if there were significant time-dependent effects of LTL on the risk of dementia at each given age. Second, depressive symptoms and the dementia-related comorbidities mentioned previously were adjusted to reduce the possibility of a potential confounder. Third, the incidence of dementia occurring within two years of recruitment was excluded to minimise the potential contribution of reverse causality to these findings in a landmark analysis. Fourth, the association between LTL and dementia was examined in people without prevalent cancer at baseline, as chemotherapy and radiotherapy for patients with cancer affect the dynamics of telomere length.^23^ Finally, the association between LTL and brain volume was re-analysed after excluding the dementia cases at baseline.

All analyses were performed using STATA V.15 statistical software (StataCorp) and R i386 V.3.4.3 (R Foundation for Statistical Computing). All p values were two‐sided, and p<0.05 was considered statistically significant.

## Results

### Baseline characteristics

Of the 439 961 participants included in this study, the mean (SD) age was 56.5 (8.1) years and the proportion of women was 54.1%. Table 1 shows the participants’ characteristics by the LTL tertile. During a median follow-up of 11.6 years, a total of 5 820 (1.3%), 1 551 (0.4%) and 767 (0.2%) individuals had developed dementia, AD and VD, respectively.

**Table 1:** Baseline characteristics by tertile of leucocyte telomere length

Characteristics	Total	LTL tertile			P value
		Lowest	Medium	Highest	
Total	439 961	147 873 (33.6)	145 712 (33.1)	146 376 (33.3)	
Sex					<0.001
Male	201 795 (45.9)	75 542 (51.1)	67 179 (46.1)	59 074 (40.4)	
Female	238 166 (54.1)	72 331 (48.9)	78 533 (53.9)	87 302 (59.6)	
Age (years), mean (SD)	56.5 (8.1)	58.3 (7.7)	56.5 (8.0)	54.7 (8.1)	<0.001
Townsend deprivation index, mean (SD)	−1.37 (3.05)	−1.38 (3.04)	−1.40 (3.04)	−1.32 (3.07)	<0.001
Ethnicity					<0.001
White	417 490 (94.9)	142 197 (96.2)	138 728 (95.2)	136 565 (93.3)	
Non-white	21 122 (4.8)	5 249 (3.5)	6 525 (4.5)	9 348 (6.4)	
Educational attainment					<0.001
College or university degree	144 997 (33.0)	44 113 (29.8)	47 746 (32.8)	53 138 (36.3)	
Professional qualifications	218 709 (49.7)	72 961 (49.3)	73 103 (50.2)	72 645 (49.6)	
Others	72 303 (16.4)	29 372 (19.9)	23 581 (16.2)	19 350 (13.2)	
Smoking status					<0.001
Never	239 906 (54.5)	76 196 (51.5)	79 798 (54.8)	83 912 (57.3)	
Former	152 996 (34.8)	54 740 (37.0)	50 429 (34.6)	47 827 (32.7)	
Current	45 687 (10.4)	16 409 (11.1)	15 058 (10.3)	14 220 (9.7)	
Alcohol frequency					<0.001
Daily or almost daily	91 079 (20.7)	32 530 (22.0)	30 184 (20.7)	28 365 (19.4)	
3–4 times/week	103 081 (23.4)	34 575 (23.4)	34 203 (23.5)	34 303 (23.4)	
1–2 times/week	113 936 (25.9)	37 686 (25.5)	37 833 (26.0)	38 417 (26.3)	
1–3 times/month	48 821 (11.1)	15 671 (10.6)	16 242 (11.1)	16 908 (11.5)	
Occasions	49 221 (11.2)	16 162 (10.9)	16 188 (11.1)	16 871 (11.5)	
Never	33 554 (7.6)	11 155 (7.5)	10 976 (7.5)	11 423 (7.8)	
BMI (kg/m^2^), mean (SD)	27.4 (4.7)	27.6 (4.7)	27.4 (4.8)	27.1 (4.8)	<0.001
Family history of dementia	58 158 (13.2)	20 276 (13.7)	19 425 (13.3)	18 457 (12.6)	<0.001
APOE ε4 carrier	124 919 (28.4)	41 587 (28.1)	41 100 (28.2)	42 232 (28.9)	<0.001
Hypertension	319 343 (72.6)	111 368 (75.3)	106 012 (72.8)	101 963 (69.7)	<0.001
Hypercholesterolaemia	217 020 (49.3)	76 824 (52.0)	72 066 (49.5)	68 130 (46.5)	<0.001
Hyperglycaemia	71 715 (16.3)	24 688 (16.7)	23 806 (16.3)	23 221 (15.9)	<0.001
Frailty phenotype					
Weight loss	67 511 (15.3)	22 922 (15.5)	22 366 (15.3)	22 223 (15.2)	0.056
Exhaustion	54 676 (12.4)	18 079 (12.2)	18 009 (12.4)	18 588 (12.7)	<0.001
Low grip strength	93 159 (21.2)	31 400 (21.2)	30 852 (21.2)	30 907 (21.1)	0.729
Physically inactive	44 747 (10.2)	15 887 (10.7)	14 552 (10.0)	14 308 (9.8)	<0.001
Slow walking pace	34 540 (7.9)	13 536 (9.2)	11 157 (7.7)	9 847 (6.7)	<0.001

Data are n (%), unless otherwise specified.

APOE, apolipoprotein E; BMI, body mass index; LTL, leucocyte telomere length; SD, standard deviation.

### LTL and dementia

**Figure 2:**
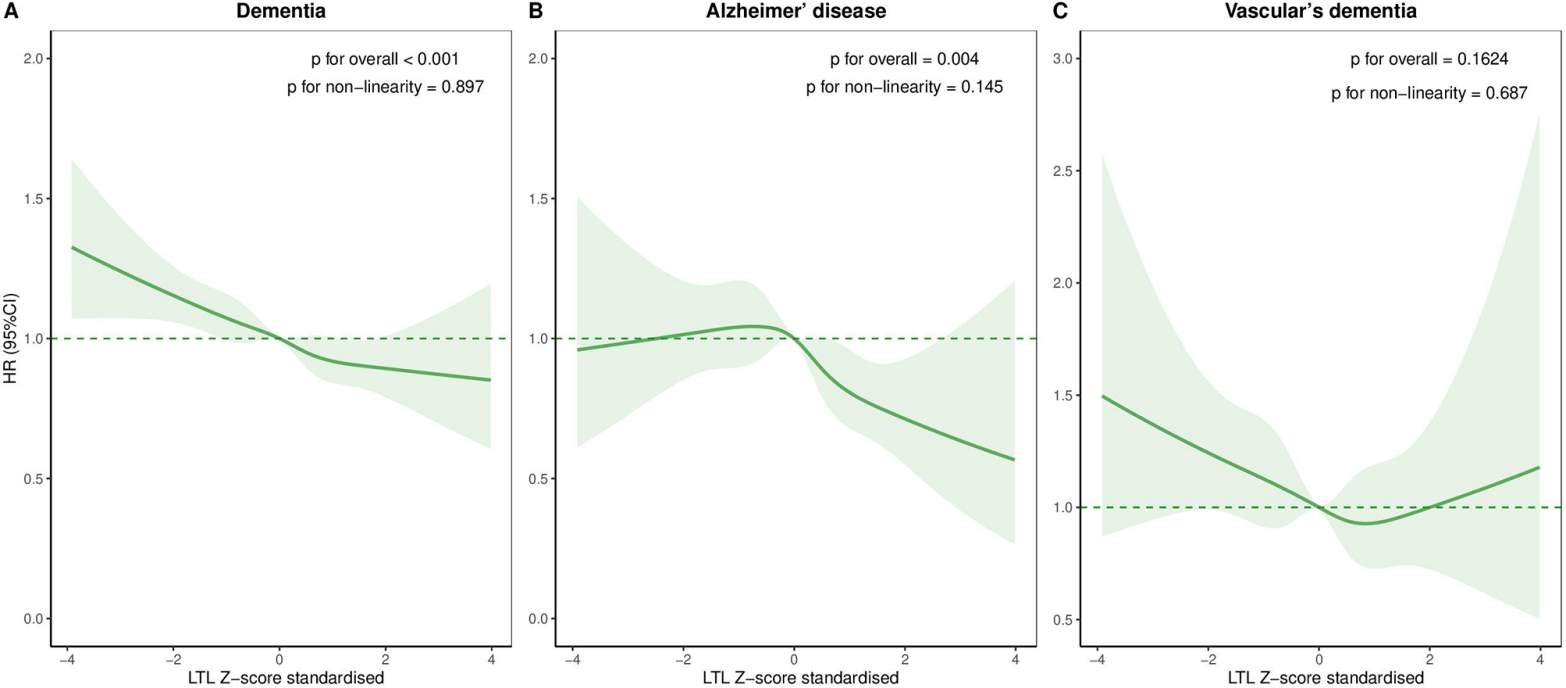
The dose-response associations of LTL with risk of incident dementia, Alzheimer’s disease and vascular dementia. Restricted cubic spline models were fitted for Cox proportional hazard models, which were adjusted for age, sex, ethnicity, educational attainment, Townsend deprivation index, smoking status, alcohol intake frequency, body mass index, APOE ε4, family history of dementia, hypertension, hypercholesterolaemia and hyperglycaemia. APOE, apolipoprotein E; CI, confidence interval; HR, hazard ratio; LTL, leucocyte telomere length.

The age-adjusted and sex-adjusted, and multivariable adjusted HRs were 1.16 (95% CI: 1.09 to 1.24) and 1.14 (95% CI: 1.06 to 1.21) for dementia, 1.30 (95% CI: 1.14 to 1.48) and 1.28 (95% CI: 1.12 to 1.46) for AD, 1.25 (95% CI: 1.04 to 1.50) and 1.18 (95% CI: 0.98 to 1.42) for VD when comparing lowest versus highest LTL tertiles (table 2). Restricted cubic spline models showed overall association of LTL with dementia and AD (p for overall<0.05), but not with VD (p for overall=0.162) (figure 2). Similar patterns of associations between LTL and dementia were found in subgroups stratified by sex (p for interaction >0.05) (online supplemental table S1). Meanwhile, these results did not appreciably alter if self-reported diabetes, self-reported CVD and depressive symptoms were further adjusted (online supplemental table S2), if participants with a diagnosis of cancer at baseline were excluded (lowest vs highest LTL: HR=1.13, 95% CI: 1.05 to 1.22 for dementia) (online supplemental table S3) or if participants within the first two years of follow-up were ruled out (lowest vs highest LTL: HR=1.06, 95% CI: 1.03 to 1.09 for dementia) (online supplemental table S4).

10.1136/gpsych-2023-101120.supp1 Supplementary data

**Table 2:** The associations of leucocyte telomere length with risks of dementia and Alzheimer’s disease using Cox proportional hazard models

	Events	Incidence rate per 1 000 person-year (95% CI)	HRs (95% CI)	
			Age and sex adjusted	Multivariable adjusted*
Dementia				
LTL tertile†				
Highest	1 368	0.80 (0.76 to 0.84)	1 (Ref.)	1 (Ref.)
Medium	1 855	1.10 (1.05 to 1.15)	1.06 (0.99 to 1.14)	1.05 (0.98 to 1.13)
Lowest	2 597	1.53 (1.47 to 1.59)	1.16 (1.09 to 1.24)	1.14 (1.06 to 1.21)
Per 1-SD reduction	5 820	1.14 (1.11 to 1.17)	1.07 (1.04 to 1.10)	1.06 (1.03 to 1.09)
Alzheimer’s disease				
LTL tertile†				
Highest	328	0.19 (0.17 to 0.21)	1 (Ref.)	1 (Ref.)
Medium	521	0.31 (0.28 to 0.34)	1.23 (1.07 to 1.41)	1.23 (1.07 to 1.42)
Lowest	702	0.41 (0.38 to 0.44)	1.30 (1.14 to 1.48)	1.28 (1.12 to 1.46)
Per 1-SD reduction	1 551	0.30 (0.29 to 0.32)	1.09 (1.03 to 1.15)	1.09 (1.03 to 1.15)
Vascular dementia				
LTL tertile†				
Highest	168	0.10 (0.08 to 0.11)	1 (Ref.)	1 (Ref.)
Medium	232	0.14 (0.12 to 0.15)	1.04 (0.85 to 1.27)	1.01 (0.83 to 1.24)
Lowest	367	0.21 (0.19 to 0.24)	1.25 (1.04 to 1.50)	1.18 (0.98 to 1.42)
Per 1-SD reduction	767	0.15 (0.14 to 0.16)	1.10 (1.02 to 1.19)	1.08 (1.00 to 1.16)

*The analyses were adjusted for age, sex, ethnicity, educational attainment, Townsend deprivation index, smoking status, alcohol intake frequency, body mass index, APOE ε4, family history of dementia, hypertension, hypercholesterolaemia and hyperglycaemia.

†LTL was categorised according to the tertile of T/S ratio. The SD of LTL was 0.131.

APOE, apolipoprotein E; CI, confidence interval; HRs, hazard ratios; LTL, leucocyte telomere length; SD, standard deviation.

We found age-dependent effects of shorter LTL on the risk of dementia, with relatively greater HRs at younger ages and effect sizes decreasing with increasing age (online supplemental figure S1). Effect modification analyses showed that the association between LTL and dementia (including AD and VD) was not modified by APOE ε4 status (p for interaction >0.05) (online supplemental table S5). However, the association between LTL and dementia was stronger in people without a family history of dementia compared with those with a family history of dementia, with the multiplicative interaction term yielding p=0.036.

### LTL and brain volume

The associations of LTL with brain volumes were examined using a subsample. In multivariable adjusted analysis, shorter LTL was associated with smaller total brain volume (β=−0.012 8, p=0.003), white matter volume (β=−0.022 4, p<0.001), hippocampus volume (β=−0.017 2, p<0.001), thalamus volume (β=−0.023 9, p<0.001), accumbens (β=−0.015 5, p=0.001), putamen (β=−0.009 4, p=0.033) and pallidum (β=−0.011 3, p=0.020) but not significantly related to grey matter, white matter hyperintensity, caudate or amygdala volume (table 3).

**Table 3:** The associations of leucocyte telomere length (per 1-SD shortening) with subcortical brain volume indicators by using linear regression models

Brain MRI (mm^3^)	Age and sex adjusted		Multivariable adjusted*	
	β (SE)	P value	β (SE)	P value
Brain atrophy				
Total brain volume	−0.133 8 (0.004 3)	0.002	−0.012 8 (0.004 2)	0.003
White matter volume	−0.021 1 (0.004 9)	<0.001	−0.022 4 (0.004 9)	<0.001
Grey matter volume	−0.002 5 (0.003 9)	0.512	−0.000 6 (0.003 8)	0.884
White matter hyperintensity	0.008 7 (0.004 8)	0.073	0.005 7 (0.004 8)	0.235
Subcortical brain volume				
Thalamus	−0.025 5 (0.004 4)	<0.001	−0.023 9 (0.004 4)	<0.001
Caudate	−0.003 7 (0.004 9)	0.455	−0.002 2 (0.004 9)	0.651
Putamen	−0.010 5 (0.004 4)	0.018	−0.009 4 (0.004 4)	0.033
Pallidum	−0.014 2 (0.004 9)	0.004	−0.011 3 (0.004 8)	0.020
Hippocampus	−0.018 0 (0.004 8)	<0.001	−0.017 2 (0.004 8)	<0.001
Amygdala	−0.003 0 (0.004 9)	0.538	−0.002 8 (0.004 9)	0.566
Accumbens	−0.016 5 (0.004 8)	0.001	−0.015 5 (0.004 8)	0.001

*Linear regression models were used and adjusted for age, sex, ethnicity, educational attainment, Townsend deprivation index, smoking status, alcohol intake frequency, body mass index, apolipoprotein E ε4, family history of dementia, hypertension, hypercholesterolaemia and hyperglycaemia. The indicators of brain volumes were converted into Z-scores.

MRI, magnetic resonance imaging; SD, standard deviation; SE, standard error.

The associations of LTL with total brain volume, white matter volume, grey matter and white matter hyperintensity volumes did not statistically differ by sex (online supplemental table S6). Additionally, there is little evidence of interaction between APOE ε4 and LTL in relation to these brain volumes (online supplemental table S7). No notable change occurred after excluding participants with prevalent dementia (total brain volume: β=−933.4, p=0.003; white matter volume: β=−904.9, p<0.001) (online supplemental table S8).

### Predictive value of LTL and brain volume

**Figure 3:**
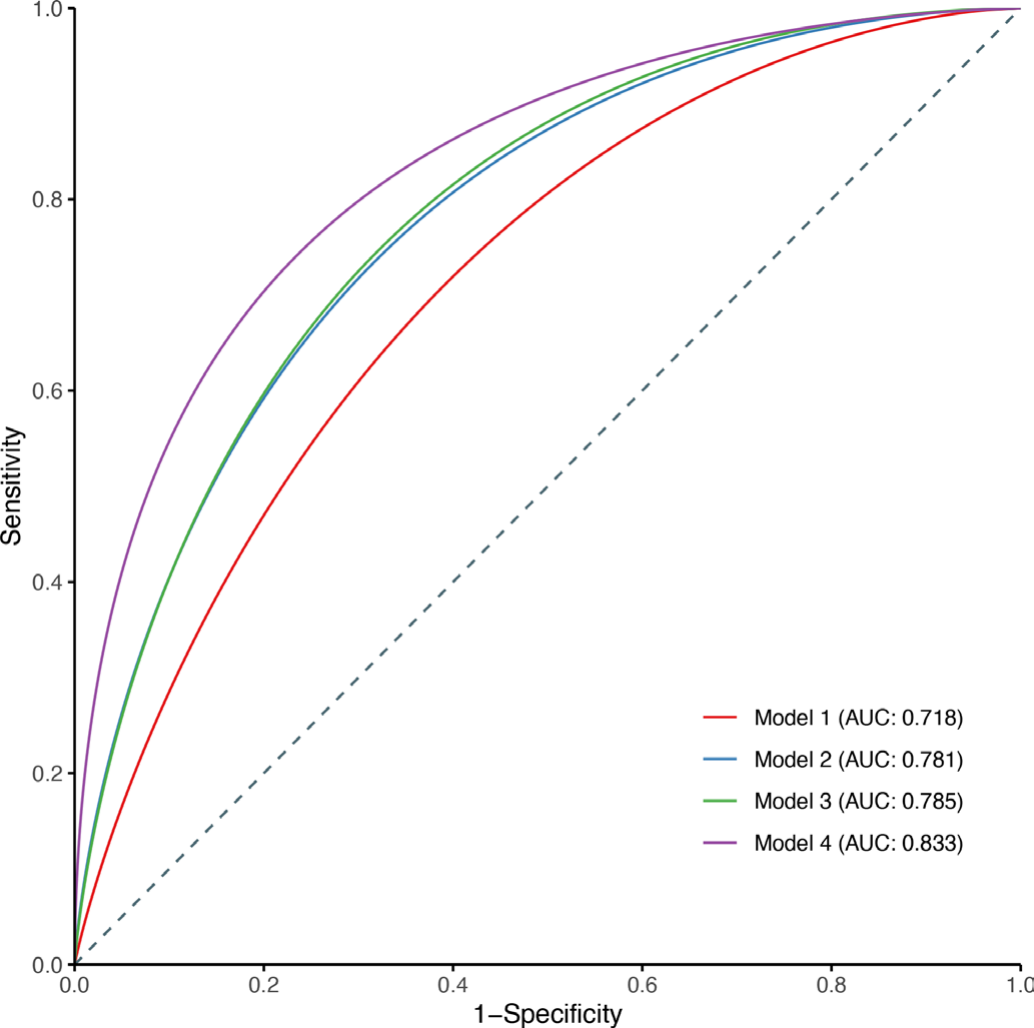
Receiver operator characteristic curves of LTL and brain volume–based models in predicting dementia. Model 1 included age, sex and cognitive function at baseline; model 2: model 1+ethnicity, educational attainment, Townsend deprivation index, smoking status, alcohol intake frequency, body mass index, APOE ε4, family history of dementia, hypertension, hypercholesterolaemia and hyperglycaemia; model 3: model 2+LTL; model 4: model 3+total brain volume, grey matter, white matter, white matter hyperintensity and hippocampus volume. APOE, apolipoprotein E; AUC, area under curve; LTL, leucocyte telomere length.

The predictive value of different models in predicting the risk of dementia is shown in figure 3. The area under curve (AUC) of model 2 that only included confounders was 0.781 (95% CI: 0.729 to 0.832). The addition of LTL to model 2 resulted in slightly improved discrimination of the model (AUC=0.785), while the difference did not reach significance (p=0.330). When we integrated several brain volumes into model 3, the predictive value was highly increased (AUC=0.833, p<0.001).

## Discussion

### Main findings

This study represents the largest longitudinal study of the association between LTL and the risk of brain health to date, taking advantage of a considerably large population that measured LTL by qPCR. We found that shorter LTL was associated with a higher risk of dementia and AD. We also observed linear associations of LTL with total brain volume, white matter volume, hippocampus, thalamus and accumbens.

The results from previous observational studies that examined the association between LTL and the risk of dementia were inconsistent with our findings. For instance, a case-control study showed that shorter LTL was not associated with a higher risk of AD.^10^ A prospective Rotterdam study including 1 961 participants demonstrated that both shorter and longer LTLs were associated with a higher risk of AD.^9^ On the other hand, a number of studies supported the association between shorter LTL and a higher risk of dementia or AD.^24 25^ One explanation for the discrepancy is attributed to the fact that LTL measurement by qPCR-based methods has high measurement error. Nonetheless, the massive LTL data generated by qPCR in the UK Biobank participants may offset the high measurement error of the method. Furthermore, our study has a very large sample size and a prospective study design, providing the strongest evidence about LTL and dementia.

The exact biological mechanisms of the observed association between LTL and dementia should be explored: those pathways that shorten telomeres, modulate the function of immune cells in the central nervous system and induce senescence of T cells in the blood.^26^ The telomere length of T cells is inversely correlated with serum levels of tumour necrosis factor-α (TNF-α) (a clinical marker of disease status). It is correlated with the proportion of CD8+T cells that lack expression of the CD28 co-stimulatory molecule, as well as correlated with apoptosis.^6^ Therefore, the telomere length of T cells correlates with AD disease severity. In addition, telomeres may play different roles in tau and amyloid pathology via multiple mechanisms.^27^ Microglial cellular senescence plays an important role in the development of AD, which is exacerbated by the presence of amyloid.^28^

A cross-sectional study that measured 1960 MRIs reported that LTL was associated with the volumes of only certain subsegmental regions, such as the hippocampus, amygdala, precuneus, thalamus and ventral diencephalon. And APOE genotypes did not substantially influence the association between LTL and brain volume.^12^ Additionally, a Swedish study including 57 midlife women revealed that shorter LTL was associated with reduced hippocampal volume, and the relationship was robust in APOE ε4 non-carriers and obscured in ε4 carriers.^29^ However, little is known about the association of LTL with white matter volume and grey matter volume. Our analysis suggested an association between LTL and white matter volume but not grey matter volume. There was no interaction between LTL and APOE ε4 allele. The underlying mechanism for the association between LTL and brain volume is unclear. Future studies should focus on how telomere shortening affects brain structure.

Our findings underscore a relationship between LTL and dementia, providing potential clinical implications. Since LTL is largely inherited, individuals who inherit shorter LTL may be predisposed to dementia,^30^ making LTL an appealing predictive biomarker for dementia. In addition, shorter LTL is widely regarded as an indicator of poorer neuropsychological condition,^6^ so measurement of LTL might be considered as an option offered to the public to motivate healthy lifestyle choices in the general population.

### Limitations

Compared with prior studies, this study is the largest single-site study of LTL that examined its association with brain volume and dementia. Our study’s strengths include a large sample size, prospective study design and the ability to adjust for potential confounders. Several limitations must be taken into account. First, we only measured telomere length in leucocytes DNA. Measurements from glial cells could have been more informative, but they were not available in large-scale studies like the UK Biobank. Whereas, a previous study showed a significant association between telomeres length measured from peripheral blood and brain tissue, which confirmed the robustness of our results.^31^ Second, LTL was measured only once at baseline in nearly 470 000 participants. Based on the results of the current study, we were unable to identify whether changes in LTL impact the chances of dementia development. Third, dementia diagnoses were obtained from electronic health records only, so some dementia cases may not have been fully covered; likewise, we inevitably omitted some undiagnosed dementia and less severe dementia cases as they might not have been mentioned in the electronic health records. However, validation studies have shown that electronic health records are reliable to ascertain dementia, with a positive predictive value of 84.5% in the UK Biobank compared with expert clinical adjudication.^32 33^ Fourth, although our analyses were adjusted for known potential biases and participants were followed up for a median of 11.8 years, it is still possible that unmeasured confounders and reverse causation remained. However, several sensitivity analyses conducted in our study supported the robustness of our findings. Finally, given the nature of an observational study design, conclusions of causality should be made with caution.

### Implications

Based on a large-scale prospective UK Biobank study, we found that LTL acts as an aging biomarker associated with the risk of dementia. Furthermore, we also observed linear associations of LTL with total and regional brain structure. These findings highlight telomere length as a potential biomarker of brain health. Further studies are needed to unravel any underlying biological pathways from LTL to dementia.

## Data Availability

Data are available upon reasonable request. The data that support the findings of this study are available from UK Biobank project site, subject to registration and application process. Further details can be found at https://www.ukbiobank.ac.uk.
