# Supplementary material for: Leucocyte telomere length, brain volume and risk of dementia: a prospective cohort study
Source: Gen Psychiatr. 2023 Sep 11;36(4):e101120. doi: 10.1136/gpsych-2023-101120 (PMC10496649; doi:10.1136/gpsych-2023-101120)
Supplement: Supplementary data [file gpsych-2023-101120supp001.pdf]

# Supplemental materials:

## Table S1: The leukocyte telomere length and dementia across sex subgroup.

| | Sex subgroup | | *P* for interaction |
|---|---|---|---|
| | Male | Female | |
| **Dementia** | | | |
| LTL tertile | | | 0.830 |
| Highest | 1 (Ref.) | 1 (Ref.) | |
| Medial | 1.07 (0.97-1.18) | 1.04 (0.94-1.15) | |
| Lowest | 1.17 (1.07-1.29) | 1.10 (1.01-1.22) | |
| Per 1-SD reduction | 1.08 (1.04-1.12) | 1.04 (1.01-1.08) | |
| **Alzheimer's disease** | | | |
| LTL tertile | | | 0.741 |
| Highest | 1 (Ref.) | 1 (Ref.) | |
| Medial | 1.32 (1.07-1.64) | 1.17 (0.97-1.40) | |
| Lowest | 1.35 (1.10-1.65) | 1.24 (1.04-1.48) | |
| Per 1-SD reduction | 1.10 (1.02-1.19) | 1.08 (1.00-1.16) | |
| **Vascular's disease** | | | |
| LTL tertile | | | 0.968 |
| Highest | 1 (Ref.) | 1 (Ref.) | |
| Medial | 1.01 (0.77-1.32) | 1.01 (0.75-1.36) | |
| Lowest | 1.20 (0.94-1.54) | 1.14 (0.87-1.51) | |
| Per 1-SD reduction | 1.08 (0.98-1.20) | 1.06 (0.94-1.20) | |

Flexible parametric survival models were used to calculate HRs, considered attained age as timescale, and adjusted for ethnicity, qualifications, Townsend deprivation index, smoking status, alcohol intake frequency, body mass index, Apolipoprotein E ε4, white blood cell count, family history of dementia, hypertension, hypercholesterolemia and hyperglycemia. LTL was categorized according to the tertiles of T/S ratio.

Abbreviations: LTL, leukocyte telomere length; SD, standard deviation.

**Table S2: The associations of leukocyte telomere length with risks of dementia and Alzheimer' disease after additional adjustment of potential confounders using Cox proportional hazards models.**

| | Model 1 † | Model 1 + self-reported diabetes | Model 1 + self-reported CVD | Model 1 + depressive symptom | Model 1 + all of three |
|---|---|---|---|---|---|
| **Dementia** | | | | | |
| LTL tertile ‡ | | | | | |
| Highest | 1 (Ref.) | 1 (Ref.) | 1 (Ref.) | 1 (Ref.) | 1 (Ref.) |
| Medial | 1.05 (0.98-1.13) | 1.05 (0.98-1.13) | 1.05 (0.98-1.12) | 1.05 (0.98-1.13) | 1.05 (0.98-1.12) |
| Lowest | 1.14 (1.06-1.21) | 1.13 (1.06-1.21) | 1.13 (1.05-1.21) | 1.14 (1.06-1.21) | 1.12 (1.05-1.20) |
| Self-reported diabetes | − | 2.05 (1.90-2.22) | − | − | 1.94 (1.79-2.10) |
| Self-reported CVD | − | − | 1.70 (1.59-1.82) | − | 1.61 (1.50-1.72) |
| Depressive symptom | − | − | − | 1.87 (1.69-2.06) | 1.77 (1.60-1.96) |
| **Alzheimer' disease** | | | | | |
| LTL tertile ‡ | | | | | |
| Highest | 1 (Ref.) | 1 (Ref.) | 1 (Ref.) | 1 (Ref.) | 1 (Ref.) |
| Medial | 1.23 (1.07-1.42) | 1.24 (1.08-1.42) | 1.23 (1.07-1.42) | 1.23 (1.07-1.42) | 1.23 (1.07-1.42) |
| Lowest | 1.28 (1.12-1.46) | 1.28 (1.12-1.46) | 1.28 (1.12-1.46) | 1.28 (1.12-1.46) | 1.27 (1.12-1.45) |
| Self-reported diabetes | − | 1.66 (1.41-1.96) | − | − | 1.61 (1.36-1.90) |
| Self-reported CVD | − | − | 1.35 (1.17-1.55) | − | 1.30 (1.13-1.49) |
| Depressive symptom | − | − | − | 1.56 (1.26-1.93) | 1.51 (1.22-1.87) |
| **Vascular' disease** | | | | | |
| LTL tertile ‡ | | | | | |
| Highest | 1 (Ref.) | 1 (Ref.) | 1 (Ref.) | 1 (Ref.) | 1 (Ref.) |
| Medial | 1.01 (0.83-1.24) | 1.02 (0.83-1.24) | 1.00 (0.82-1.23) | 1.01 (0.82-1.24) | 1.00 (0.81-1.23) |
| Lowest | 1.18 (0.98-1.42) | 1.17 (0.97-1.41) | 1.16 (0.96-1.39) | 1.18 (0.97-1.43) | 1.14 (0.94-1.39) |
| Self-reported diabetes | | 2.76 (2.29-3.32) | | | 2.48 (2.04-3.03) |
| Self-reported CVD | | | 2.57 (2.18-3.02) | | 2.36 (1.98-2.80) |
| Depressive symptom | | | | 2.26 (1.76-2.91) | 2.03 (1.58-2.62) |

† Flexible parametric survival models were used to calculate HRs, considered attained age as timescale, and adjusted for sex, ethnicity, qualifications, Townsend deprivation index, smoking status, alcohol intake frequency, body mass index, Apolipoprotein E ε4, white blood cell count,

family history of dementia, hypertension, hypercholesterolemia.

‡ LTL was categorized according to the tertile of T/S ratio. The SD of LTL was 0.131.

Abbreviations: LTL, leukocyte telomere length.

**Table S3: The associations of leukocyte telomere length with risks of dementia and Alzheimer' disease after excluding prevalent cancer at baseline using Cox proportional hazards models.**

| | Events | Incidence rate per 1000 person-year | Hazard ratio | |
|---|---|---|---|---|
| | | | Model 1 * | Model 2 † |
| **Dementia** | | | | |
| LTL tertile | | | | |
| Highest | 1206 | 0.77 (0.73-0.81) | 1 (Ref.) | 1 (Ref.) |
| Medial | 1638 | 1.06 (1.01-1.11) | 1.05 (0.98-1.14) | 1.05 (0.97-1.13) |
| Lowest | 2299 | 1.48 (1.42-1.55) | 1.165 (1.08-1.25) | 1.13 (1.05-1.22) |
| Per 1-SD reduction | 5143 | 1.10 (1.07-1.13) | 1.07 (1.04-1.10) | 1.06 (1.03-1.09) |
| **Alzheimer' disease** | | | | |
| LTL tertile | | | | |
| Highest | 292 | 0.18 (0.16-0.21) | 1 (Ref.) | 1 (Ref.) |
| Medial | 459 | 0.29 (0.27-0.32) | 1.21 (1.04-1.40) | 1.22 (1.05-1.41) |
| Lowest | 628 | 0.40 (0.37-0.44) | 1.30 (1.13-1.49) | 1.28 (1.11-1.47) |
| Per 1-SD reduction | 1379 | 0.29 (0.28-0.31) | 1.09 (1.03-1.15) | 1.08 (1.02-1.15) |
| **Vascular's disease** | | | | |
| LTL tertile | | | | |
| Highest | 146 | 0.09 (0.08-0.11) | 1 (Ref.) | 1 (Ref.) |
| Medial | 208 | 0.13 (0.12-0.15) | 1.06 (0.86-1.31) | 1.04 (0.84-1.28) |
| Lowest | 322 | 0.21 (0.18-0.23) | 1.24 (1.02-1.51) | 1.18 (0.97-1.44) |
| Per 1-SD reduction | 676 | 0.14 (0.13-0.16) | 1.09 (1.01-1.19) | 1.07 (0.98-1.16) |

* Flexible parametric survival models were used to calculate HRs, considered attained age as timescale, and adjusted for sex.

† Flexible parametric survival models were used to calculate HRs, considered attained age as timescale, and adjusted for sex, ethnicity, qualifications, Townsend deprivation index, smoking status, alcohol intake frequency, body mass index, Apolipoprotein E ε4, white blood cell count, family history of dementia, hypertension, hypercholesterolemia and hyperglycemia.

Abbreviations: LTL, leukocyte telomere length; SD, standard deviation.

**Table S4: The associations of leukocyte telomere length with risks of dementia and Alzheimer' disease after excluding first two years of dementia cases during follow-up time.**

| | Events | Incidence rate per 1000 person-year | Hazard ratio | |
|---|---|---|---|---|
| | | | Model 1 * | Model 2 † |
| **Dementia** | | | | |
| LTL tertile | | | | |
| Highest | 1330 | 0.78 (0.74-0.82) | 1 (Ref.) | 1 (Ref.) |
| Medial | 1810 | 1.07 (1.02-1.12) | 1.06 (0.98-1.13) | 1.05 (0.98-1.13) |
| Lowest | 2522 | 1.49 (1.43-1.54) | 1.15 (1.08-1.23) | 1.12 (1.05-1.20) |
| Per 1-SD reduction | 5662 | 1.11 (1.08-1.14) | 1.07 (1.04-1.10) | 1.06 (1.03-1.09) |
| **Alzheimer' disease** | | | | |
| LTL tertile | | | | |
| Highest | 325 | 0.19 (0.17-0.21) | 1 (Ref.) | 1 (Ref.) |
| Medial | 518 | 0.31 (0.28-0.33) | 1.23 (1.07-1.42) | 1.24 (1.08-1.42) |
| Lowest | 698 | 0.41 (0.38-0.42) | 1.30 (1.14-1.48) | 1.29 (1.13-1.47) |
| Per 1-SD reduction | 1541 | 0.30 (0.29-0.32) | 1.09 (1.03-1.15) | 1.09 (1.03-1.15) |
| **Vascular's disease** | | | | |
| LTL tertile | | | | |
| Highest | 165 | 0.10 (0.08-0.11) | 1 (Ref.) | 1 (Ref.) |
| Medial | 227 | 0.13 (0.12-0.15) | 1.03 (0.84-1.26) | 1.01 (0.82-1.23) |
| Lowest | 359 | 0.21 (0.19-0.23) | 1.23 (1.02-1.49) | 1.17 (0.97-1.41) |
| Per 1-SD reduction | 751 | 0.15 (0.14-0.16) | 1.10 (1.01-1.19) | 1.07 (0.99-1.16) |

* Flexible parametric survival models were used to calculate HRs, considered attained age as timescale, and adjusted for sex.

† Flexible parametric survival models were used to calculate HRs, considered attained age as timescale, and adjusted for sex, ethnicity, qualifications, Townsend deprivation index, smoking status, alcohol intake frequency, body mass index, Apolipoprotein E ε4, white blood cell count, family history of dementia, hypertension, hypercholesterolemia and hyperglycemia.

Abbreviations: LTL, leukocyte telomere length; SD, standard deviation.

**Table S5: The effect modification of familial factors on the associations between leukocyte telomere length and dementia.**

| | APOE ε4 gene | | Family history of dementia | |
|---|---|---|---|---|
| | Carrier | Non-carrier | With | Without |
| **Dementia** | | | | |
| LTL tertile | | | | |
| Highest | 1 (Ref.) | 1 (Ref.) | 1 (Ref.) | 1 (Ref.) |
| Medial | 1.08 (0.98-1.20) | 1.03 (0.93-1.14) | 1.21 (1.04-1.42) | 1.02 (0.94-1.10) |
| Lowest | 1.14 (1.04-1.25) | 1.14 (1.04-1.25) | 1.14 (0.98-1.32) | 1.14 (1.06-1.23) |
| P for interaction | 0.624 | | 0.036 | |
| **Alzheimer's disease** | | | | |
| LTL tertile | | | | |
| Highest | 1 (Ref.) | 1 (Ref.) | 1 (Ref.) | 1 (Ref.) |
| Medial | 1.25 (1.05-1.49) | 1.21 (0.97-1.52) | 1.27 (0.97-1.65) | 1.22 (1.04-1.44) |
| Lowest | 1.32 (1.12-1.56) | 1.22 (0.98-1.51) | 1.26 (0.97-1.63) | 1.29 (1.11-1.51) |
| P for interaction | 0.865 | | 0.870 | |
| **Vascular's disease** | | | | |
| LTL tertile | | | | |
| Highest | 1 (Ref.) | 1 (Ref.) | 1 (Ref.) | 1 (Ref.) |
| Medial | 0.96 (0.73-1.27) | 1.07 (0.80-1.42) | 0.79 (0.49-1.26) | 1.07 (0.86-1.33) |
| Lowest | 1.11 (0.86-1.44) | 1.26 (0.97-1.64) | 1.15 (0.76-1.73) | 1.19 (0.97-1.46) |
| P for interaction | 0.773 | | 0.420 | |

All models considered attained age as timescale, and adjusted for sex, ethnicity, qualifications, Townsend deprivation index, smoking status, alcohol intake frequency, body mass index, Apolipoprotein E ε4, family history of dementia, white blood cell count, hypertension, hypercholesterolemia and hyperglycemia. LTL was categorized according to the tertiles of T/S ratio.

Abbreviations: APOE ε4, Apolipoprotein E ε4; LTL, leukocyte telomere length

**Table S6: Interaction between leukocyte telomere length and sex on brain volume.**

| | White matter volume | | Gray matter volume | | Total brain volume | | White matter hyperintensity volumes | |
|---|---|---|---|---|---|---|---|---|
| | β (95% CI) | P | β (95% CI) | P | β (95% CI) | P | β (95% CI) | P |
| LTL tertile | | | | | | | | |
| Highest | 0 (Ref.) | | 0 (Ref.) | | 0 (Ref.) | | 0 (Ref.) | |
| Medial | -2318.6 (-3634.3, -1002.8) | 0.001 | 372.8 (-840.2, 1585.9) | 0.547 | 60.7 (-152.0, 273.6) | 0.576 | -1945.7 (-3981.9, 90.5) | 0.061 |
| Lowest | -903.8 (-2171.7, 364.1) | 0.162 | 631.5 (-537.4, 1800.4) | 0.290 | -73.1 (-278.1, 131.9) | 0.485 | -272.4 (-2234.5, 1689.7) | 0.786 |
| Sex | 9725.7 (8344.9, 11106.5) | | -22145.5 (-23418.5, -20872.4) | | 68.3 (-154.9, 291.5) | 0.549 | -12429.8 (-14556.6, -10283.0) | |
| Interaction | | | | | | | | |
| Medial LTL tertile * male | 811.7 (-1099.3, 2722.7) | 0.405 | -1639.6 (-3401.7, 121.9) | 0.068 | 182.5 (-126.4, 491.5) | 0.247 | -828.1 (-3785.5, 2129.2) | 0.583 |
| Lowest LTL tertile * male | 26.3 (-1879.5, 1932.1) | 0.978 | -1442.69 (-3199.6, 314.4) | 0.108 | 322.3 (14.1, 630.4) | 0.040 | -1416.1 (-4365.4, 1533.2) | 0.347 |
| LTL (continuous) | | | | | | | | |
| LTL | -1043.3 (-1576.0, -510.6) | <0.001 | 329.7 (-161.4, 820.9) | 0.188 | -713.6 (-1538.0, 110.9) | 0.09 | -1.64 (-87.78, 84.51) | 0.97 |
| Sex | 11837.9 (6774.1, 16901.8) | <0.001 | -28201.6 (-32870.9, -23532.9) | <0.001 | -16363.7 (-24200.8, -8526.7) | | 806.4 (-12.5, 1625.3) | 0.054 |
| Interaction | 282.4 (-499.9, 1064.7) | 0.479 | -781.7 (-1503.1, -60.4) | 0.034 | -499.3 (-1710.0, 711.5) | 0.419 | 88.4 (-38.1, 214.9) | 0.171 |

Linear regression models were used and adjusted for age, ethnicity, qualifications, Townsend deprivation index, smoking status, alcohol intake frequency, body mass index, Apolipoprotein E ε4, family history of dementia, white blood cell count, hypertension, hypercholesterolemia and hyperglycemia.

Abbreviations: LTL, leukocyte telomere length.

BMJ Publishing Group Limited (BMJ) disclaims all liability and responsibility arising from any reliance placed on this supplemental material which has been supplied by the author(s)

**Table S7: Interaction between leukocyte telomere length and APOE ε4 on brain volume.**

| | White matter volume | | Gray matter volume | | Total brain volume | | White matter hyperintensity volumes | |
|---|---|---|---|---|---|---|---|---|
| | **β (95% CI)** | **P** | **β (95% CI)** | **P** | **β (95% CI)** | **P** | **β (95% CI)** | **P** |
| LTL tertile | | | | | | | | |
| Highest | 0 (Ref.) | | 0 (Ref.) | | 0 (Ref.) | | 0 (Ref.) | |
| Medial | -1884.2 (-33014.7, -753.7) | 0.001 | -576.3 (-1618.7, 466.1) | 0.279 | -2460.6 (-4210.1, -711.0) | 0.006 | 153.2 (-29.7, 336.2) | 0.101 |
| Lowest | -1074.6 (-2191.6, 42.3) | 0.059 | -86.4 (-1116.4, 943.6) | 0.869 | -1161.1 (-2889.8, 567.6) | 0.188 | 123.1 (-57.6, 303.9) | 0.182 |
| APOE 4 carrier | -299.3 (-1792.4, 1193.8) | 0.694 | -600.2 (-1977.1, 776.6) | 0.393 | -899.6 (-3210.4, 1411.2) | 0.445 | 266.5 (24.9, 508.1) | 0.031 |
| Interaction | | | | | | | | |
| Medial LTL tertile * APOE 4 carrier | -198.8 (-2308.5, 1910.9) | 0.853 | 797.4 (-1147.9, 2742.8) | 0.422 | 598.7 (-2666.3, 3863.7) | 0.719 | -62.0 (-403.4, 279.4) | 0.722 |
| Lowest LTL tertile * APOE 4 carrier | 539.1 (-1572.3, 2650.5) | 0.617 | 226.1 (-1720.9, 2173.0) | 0.820 | 765.2 (-2502.4, 4032.9) | 0.646 | -195.8 (-537.5, 145.9) | 0.261 |
| LTL (continuous) | | | | | | | | |
| LTL | -917.7 (-1383.4, -452.1) | | -47.4 (-476.8, 382.0) | 0.829 | -965.2 (-1685.9, -244.4) | 0.009 | 57.6 (-17.8, 132.9) | 0.134 |
| APOE 4 carrier | -123.1 (-5702.6, 5456.4) | 0.966 | 373.6 (-4771.7, 5519.0) | 0.887 | 250.7 (-8384.9, 8886.2) | 0.955 | 247.4 (-1150.4, 655.6) | 0.591 |
| Interaction | 12.4 (-846.4, 871.1) | 0.977 | 99.0 (-692.9, 890.9) | 0.806 | 111.4 (-1217.7, 144.6) | 0.869 | -66.8 (-205.8, 72.1) | 0.346 |

Linear regression models were used and adjusted for age, sex, ethnicity, qualifications, Townsend deprivation index, smoking status, alcohol intake frequency, body mass index, family history of dementia, white blood cell count, hypertension, hypercholesterolemia and hyperglycemia.

Abbreviations: LTL, leukocyte telomere length; APOE 4, Apolipoprotein E ε4.

**Table S8: β-Coefficients and 95% confidence intervals (CIs) for the association between leukocyte telomere length (LTL) and brain volume after excluding prevalent dementia at baseline.**

| Brain volume ($mm^3$) | Multivariate adjusted * | | |
|---|---|---|---|
| | β | (95% CI) | P value |
| **Total brain volume** | | | |
| Highest LTL tertile | 0 (Ref.) | 0 (Ref.) | |
| Medial LTL tertile | -852.8 | -2424.6, 518.9 | 0.204 |
| Lowest LTL tertile | -2318.7 | -3812.2, -825.2 | 0.002 |
| Per 1-SD reduction of LTL | -933.4 | -1546.1, -320.7 | 0.003 |
| **White matter volume** | | | |
| Highest LTL tertile | 0 (Ref.) | 0 (Ref.) | |
| Medial LTL tertile | -942.6 | -1893.4, 8.1 | 0.052 |
| Lowest LTL tertile | -1930.7 | -2895.5, -965.8 | <0.001 |
| Per 1-SD reduction of LTL | -904.9 | -1300.7, -509.1 | <0.001 |
| **Grey matter volume** | | | |
| Highest LTL tertile | 0 (Ref.) | 0 (Ref.) | |
| Medial LTL tertile | -10.2 | -887.1, 866.7 | 0.982 |
| Lowest LTL tertile | -388.0 | -1277.9, 501.8 | 0.393 |
| Per 1-SD reduction | -28.5 | -393.6, 336.6 | 0.878 |
| **White matter hyperintensity volume** | | | |
| Highest LTL tertile | 0 (Ref.) | 0 (Ref.) | |
| Medial LTL tertile | 66.6 | -86.5, 219.6 | 0.394 |
| Lowest LTL tertile | 126.7 | -28.6, 282.0 | 0.110 |
| Per 1-SD reduction of LTL | 38.2 | -25.5, 101.9 | 0.240 |

*Linear regression models adjusted for age, sex, ethnicity, qualifications, Townsend deprivation index, smoking status, alcohol intake frequency, body mass index, Apolipoprotein E ε4, family history of dementia, white blood cell count, hypertension, hypercholesterolemia and hyperglycemia.

Abbreviations: LTL, leukocyte telomere length; SD, standard deviation.

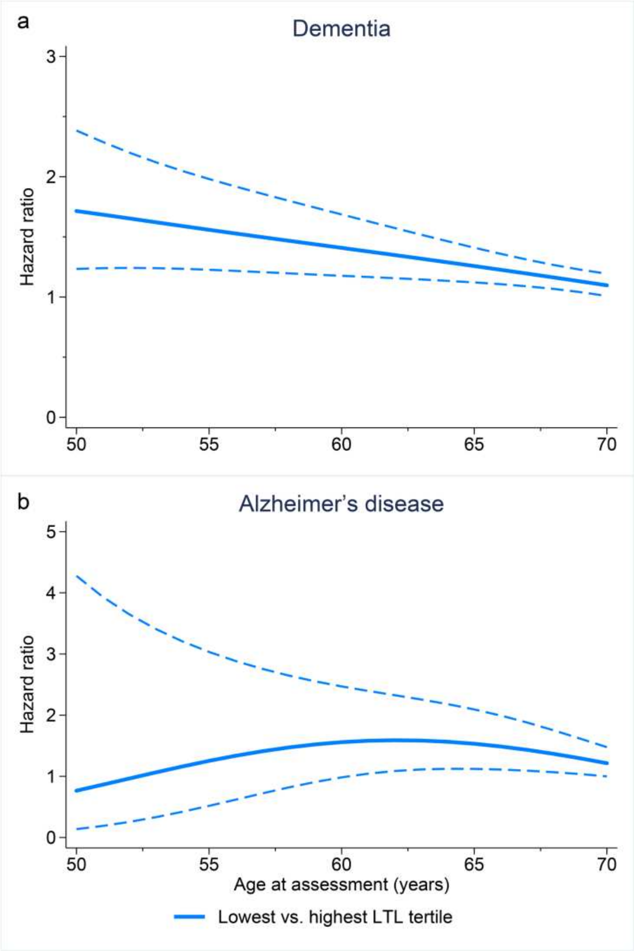


**Figure S1: Age-dependent associations of leukocyte telomere length with risks of dementia and Alzheimer' disease.**

Flexible parametric survival models with leukocyte telomere length (LTL) as time-dependent variable were used to calculate HRs, considered attained age as timescale, and adjusted for sex, ethnicity, qualifications, Townsend deprivation index, smoking status, alcohol intake frequency, body mass index, Apolipoprotein E ε4, family history of dementia, white blood cell count, hypertension, hypercholesterolemia and hyperglycemia. LTL was categorized into according to the tertiles of T/S ratio.
